# Exogenous GABA regulates the growth traits, photosynthesis, antioxidant properties, and nitrogen metabolism in *Isatis indigotica* Fortune seedlings

**DOI:** 10.3389/fpls.2026.1749021

**Published:** 2026-02-11

**Authors:** Siren Cheng, Pingshan Fan, Pengpeng He, Keying Mei, Hongchao Liu, Kang Sun, Yong Ren

**Affiliations:** 1 College of Biological Sciences and Technology, Yili Normal University, Yining, China; 2 Key Laboratory of Plant Resources Protection and Utilization in Xinjiang Yili Valley, Yili Normal University, Yining, China; 3 Sanya Institute of Breeding and Multiplication, Hainan University, Sanya, Hainan, China

**Keywords:** growth and development, *Isatis indigotica* Fortune, physiological and biochemical parameters, seedlings, γ-aminobutyric acid

## Abstract

To investigate the effects of exogenous γ-aminobutyric acid (GABA) application on the growth, development, and related parameters associated with different physiological and biochemical processes of *Isatis indigotica* Fortune (*I. indigotica*) seedlings, a pot experiment was conducted. Seedlings were collected from two provenance areas (Gansu and Hebei) and treated at the six-leaf stage with four GABA concentrations: 0 (CK), 2.5 (T1), 5 (T2), and 7.5 (T3) mM·L^-1^. Compared with CK, T1 and T2 treatments improved plant height, total fresh mass, and total dry mass at all sampling stages, with increments of 0.99%-7.86%, 2.99%-12.79%, and 1.27%-6.90%, respectively. These promotional effects were attributed to increased photosynthetic capacity, pigment contents, and activities of nitrogen metabolism enzymes. Meanwhile, root morphology was also promoted by T1 and T2 treatments. In contrast, inhibitory effects of T3 treatment on plant height, total biomass accumulation, and root morphology were also observed. Furthermore, exogenous treatments T1 and T2 markedly elevated the antioxidant capacity of *I. indigotica* seedlings, as evidenced by the increased activities of superoxide dismutase (SOD), peroxidase (POD), and catalase (CAT), along with the enhanced soluble protein content in both leaves and roots. Meanwhile, these two treatments significantly reduced the accumulation of malondialdehyde (MDA) and hydrogen peroxide (H_2_O_2_) in the aboveground and underground tissues of the seedlings. Structural equation modeling (SEM) further revealed that the total antioxidant capacity exerted positive regulatory effects on plant height, total biomass accumulation, and root morphological traits through both direct and indirect pathways. Principal component analysis (PCA) demonstrated that T2 treatment induced the most prominent variation in seedling growth and physiological traits. Specifically, glutamate synthase (GOGAT) activity showed the strongest correlation with the first principal component (PC1), while POD activity was most closely associated with the second principal component (PC2). Collectively, these findings suggest that 5 mM·L^-1^ is the optimal concentration of exogenous GABA for the cultivation of *I. indigotica* seedlings.

## 1 Introduction

*Isatis indigotica* Fortune (*I. indigotica*) is a biennial herbaceous plant belonging to the Cruciferae family, a taxonomic group that encompasses over 330 known genera and 3,700 species (Su et al., 2025). In traditional Chinese medicine, *I. indigotica* possesses prominent medicinal properties, specifically the abilities to clear heat, resolve toxicity, cool the blood, soothe sore throats, and reduce swelling (He et al., 2022; Su et al., 2025). Concurrently, numerous modern scientific studies have documented its notable pharmacological activities in modern medicine, including antimicrobial, antiviral, antiendotoxin, anticancer, and immunomodulatory effects (Chen et al., 2021; He et al., 2022; Li et al., 2022). Owing to its significant contributions to clinical therapy, *I. indigotica* is widely cultivated and highly valued in China, with its primary distribution spanning provinces such as Xinjiang, Gansu, Hebei, Henan, Guizhou, Anhui, and Heilongjiang (He et al., 2022; Su et al., 2025).

Prior works have indicated the growth and development of *I. indigotica* could be affected by various environmental changes and the application of different exogenous substances. For instance, He et al. (2022) found that the medium nitrogen application (200 kg·ha^-1^) coupled with a moderate irrigation level (maintaining the soil moisture content at 70-80%) could improve both the yield production and medicinal components accumulation in *I. indigotica*. It is worth emphasizing that the successful establishment of the seedling stage lays a fundamental and pivotal foundation for the plant’s subsequent vegetative growth, reproductive development, yield formation, and accumulation of medicinal components. Li et al. (2017) reported that when the CO_2_ concentration in the growth environment was elevated from 385 to 590 μmol·mol^-1^, parameters related to photosynthesis, including net photosynthetic rate (Pn), transpiration rate (Tr), and stomatal conductance (Gs), were increased, as were the weight of leaves, roots, and whole plants of *I. indigotica* at 49 days after sowing. Moreover, foliar application of 500 μM methyl jasmonate to 80-day-old *I. indigotica* seedlings induced the promotion of bioactive compound contents [e.g., soluble protein, soluble sugar, and lignin) and antioxidant enzyme activities (e.g., catalase (CAT), peroxidase (POD), and superoxide dismutase (SOD)] (Liu et al., 2022). Similarly, the study of Jiang et al. (2020) discovered that seed priming with 15 g·L^-1^ CaCl_2_, 0.2 g·L^-1^ GA_3_, and 40 mM hydrogen peroxide (H_2_O_2_) respectively enhanced the salt tolerance of *I. indigotica* seedlings.

Gamma-aminobutyric acid (γ-aminobutyric acid, GABA) is a ubiquitous four-carbon amino acid that does not participate in protein synthesis, and it is widely present in both plants and animals (Li et al., 2016, 2021). Since GABA was first isolated from potato tubers in 1949 and subsequently identified in the human brain in 1950, numerous researchers have gradually uncovered its specific regulatory effects on living organisms (Spiering, 2018; Li et al., 2021). These effects span multiple biological processes, including safeguarding human health, enhancing plant defense, regulating osmotic pressure, modulating metabolic activities, facilitating nutrient storage, and mediating signal transduction (Li et al., 2016; Mills, 2021; Shelp et al., 2021; Xu et al., 2021; Almutairi et al., 2024; Sulieman et al., 2024).

In the field of plant physiology research, many previous works have revealed the effect of exogenous GABA application on plant growth and development under both stress and non-stress conditions, such as drought, salinity, heavy metal, and nutrition deficiency (Zhou et al., 2021; Aljuaid and Ashour, 2022; Sun et al., 2023; Yu et al., 2024; Geng et al., 2025). While GABA is rightly recognized as a crucial stress metabolite, its physiological role extends beyond stress responses. Accumulating evidence indicates that GABA is fundamentally involved in regulating core physiological processes under normal growth conditions, such as carbon-nitrogen metabolism and signal transduction. This broader functional scope underscores its potential as a biostimulant for improving crop performance even in the absence of environmental stress. For example, Li et al. (2016) found that foliar spraying of GABA increased photosynthetic capacity, antioxidant enzyme activities, and nitrogen metabolism, ultimately promoting maize seedling growth. Similarly, applications of GABA have been shown to improve grain quality and antioxidant attributes in fragrant rice, as well as enhance fruit quality in tomatoes (Gao et al., 2020; Xie W. et al., 2020; Wu X. et al., 2024). For medicinal plants like *I. indigotica*, optimizing seedling vigor during standard cultivation is a key agronomic objective. However, exogenous GABA application has rarely been studied regarding its effects on the growth of *I. indigotica* seedlings, nor has its potential influence on the various related parameters involved in different physiological and biochemical processes been well documented. Furthermore, the six-leaf stage was selected for intervention as it represents a pivotal period for the establishment of autotrophic growth, where modulating central metabolism can profoundly impact seedling vigor and development.

Hence, a pot experiment with four GABA concentration levels (0, 2.5, 5, and 7.5 mM·L^-1^) applied at the six-leaf stage was conducted in this study. The objectives of the study were as follows: (1) To evaluate the photosynthetic capacity by determining the Pn, Gs, Tr, intercellular CO_2_ concentration (Ci) and pigment contents; (2) To determine the contents of resistance-related substances and the activities of resistance-related enzymes, including malondialdehyde (MDA), H_2_O_2_, soluble protein, CAT, POD, and SOD; (3) To investigate the regulatory effect of exogenous GABA application at the six-leaf stage on the activities of nitrogen metabolism enzymes in *I. indigotica*; (4) To assess the biomass accumulation by measuring the fresh mass and dry mass of the whole *I. indigotica* plant at each sampling stage.

## 2 Materials and methods

### 2.1 Plant materials and growth conditions

Representative *I. indigotica* seeds from two key provenance areas (Gansu and Hebei) were selected, as they exhibit superior medicinal quality and a leading yield. Prior to sowing, the seeds of two provenance areas were disinfected by 6% sodium hypochlorite solution for 15 min, followed by three rinses with distilled water. For the seeds germination, the sterilized seeds were firstly soaked in distilled water at room temperature for 12h, subsequently covered with double-layer absorbent paper fully filled with water, and then incubated in a growth chamber at 30°C for 72h. The germinated seeds were manually sown into seedling-raising pots (8 cm × 8 cm) filled with 115 g of dry mixed nutrient stroma, at a depth of 1.5 cm with three seeds per pot. The pots were then placed in an illumination incubator under controlled conditions: temperature 26°C, relative humidity 80%, full illumination intensity, and a 12-hour light period. Notably, the mixed nutrient stroma consisted of nutrient soil and coconut bran (both purchased from a local horticultural market) at a 2:1 mass ratio. The detailed nutrient properties of mixed nutrient stroma were detected by Guangdong Detection Center of Microbiology (Guangzhou, Guangdong) and presented as follow: 1.23% of total nitrogen, 0.12% of total phosphorous, 1.99% of total potassium, 1.75 g·kg^-1^ of available nitrogen, 79.60 mg·kg^-1^ of available phosphorous, 3.04 g·kg^-1^ available potassium, 274.00 g·kg^-1^ of organic matter, and a pH of 7.23.

### 2.2 Experiment design and plant sampling

At the six-leaf stage, four GABA solutions of different concentrations (0, 2.5, 5, and 7.5 mM·L-1) were selected to establish a dose-response gradient based on references to existing literature on GABA application in plants (Cao et al., 2024; Yu et al., 2024), each mixed with 1.25‰ Tween-80 and noted as CK, T1, T2, and T3 respectively, were evenly sprayed onto the leaves, with one application per day for three consecutive days. Each spraying session was continued until the solution drop-lets on the leaves were about to fall off. A complete randomized design was carried out for the experiments in this study, and each treatment contained 40 pots to ensure an adequate supply of *I. indigotica* seedlings for sampling throughout the entire experiment process.

Three sampling stages were set at 1 day, 3 days and 5 days after spraying, named as 1d AF, 3d AF, and 5d AF. At each sampling stage, 8–10 representative *I. indigotica* seedlings were carefully uprooted from the pots, rinsed with distilled water, and immediately separated into leaves and roots. These tissues were then immersed in liquid nitrogen for 15 seconds and subsequently stored in an ultra-low temperature freeze at -80°C for the determination of physiological and biochemistry parameters.

### 2.3 Determination of photosynthetic capacity

Following the method described of Cheng et al (Cheng et al., 2022), during all experimental stages, eight representative *I. indigotica* seedlings were selected in each treatment, and their fully expanded leaves were used to determine photosynthetic activity. Measurements were conducted using a portable photosynthetic fluorometer (Yaxin-1105, Yaxin, Beijing) between 9:00 and 11:00 AM. Before detection, the device should be preheated for 20 minutes and complete its self-inspection. The leaf chamber had a specification of 25 cm × 25 cm, and the flow rate was set at 0.6 mM·s^-1^.

### 2.4 Determination of plant height and total biomass accumulation

After determining the photosynthetic activity, an additional eight representative *I. indigotica* seedlings from each treatment were dug out, thoroughly washed with distilled water three times, and immediately blotted dry with absorbent paper to measure their plant height. These seedlings were then weighed, and their total fresh weight was recorded as total fresh mass (TFM). Subsequently, the seedlings were dried in an oven at 80°C until a constant weight was achieved, and the total dry weight of the seedlings was recorded as total dry mass (TDM) (Cheng et al., 2022).

### 2.5 Determination of root morphology

Consistent with the method described by Cheng et al (Cheng et al., 2022), at each sampling stage, four representative *I. indigotica* seedlings were selected from per treatment. The roots were separated, rinsed with distilled water three times to remove surface stroma, and then spread flat on a root system imaging disk. Root morphological indicators were scanned and analyzed using a root analysis instrument (GXY-A plus, Top Cloud Agri, Zhejiang).

### 2.6 Determination of antioxidant ability and pigment contents

The pigment (chlorophyll a, chlorophyll b and carotenoid) contents determinations were according to the method of Wu et al. (Wu Y. et al., 2024). About 0.3g leaves were soaked in 10 ml 95% ethanol solution for 12h. the extracting solution was then measured using a microplate reader (Epoch, Bio-tek, USA) at absorbance wavelengths of 470, 649, and 665 nm, with the results expressed as mg·g^-1^ FW.

With the description of method published by Wu et al. (Wu Y. et al., 2024), the preparation process of extracted solution for antioxidant ability measurement was as follow: approximately 0.3 g of leaves and roots was ground into a powder using liquid nitrogen, after which 6 mL of phosphate buffer solution (pH = 7.8) was added. The mixture was left to stand in a refrigerator at 4°C for 12 hours and then centrifuged under 12000 r·min^-1^ for 5 min at 4°C.

The SOD activity was determined based on its ability to inhibit the photochemical reduction of nitroblue tetrazolium (NBT) by superoxide radicals generated from a riboflavin-light system, with one unit (U) of SOD activity defined as the amount of enzyme required to cause 50% inhibition of NBT reduction at 560 nm, and expressed as U·g^-1^ FW. POD activity was assayed by monitoring the oxidation of guaiacol in the presence of H_2_O_2_, which produces a brownish product with an increasing absorbance at 470 nm, and expressed as U·g^-1^·min^-1^ FW. CAT activity was measured by tracking the decomposition of H_2_O_2_, which results in a decrease in absorbance at 240 nm, and expressed as U·g^-1^·min^-1^ FW.

The H_2_O_2_ content was quantified using the titanium sulfate method, where H_2_O_2_ reacts with titanium ions to form a yellow peroxo-complex measured at 410 nm, and the units were noted as μmol·g^-1^ FW. Soluble protein content was estimated according to the method of Bradford using Coomassie Brilliant Blue G-250 dye. The dye binds to proteins under acidic conditions, causing a shift in absorbance measured at 595 nm. The protein concentration was determined by comparing the absorbance with a standard curve prepared using bovine serum albumin (BSA), and the results are expressed as μg per gram fresh weight (μg·g^-1^ FW).

The MDA content, an indicator of lipid peroxidation, was determined via the thiobarbituric acid (TBA) reaction, where MDA reacts with TBA to form a red adduct with maximum absorbance at 532 nm (with corrections for non-specific absorbance at 600 and 450 nm) with the results expressed as μmol·g^-1^ FW.

### 2.7 Determination of enzymes activities related to nitrogen metabolism

According to the methods described by Li et al (Li et al., 2016), the enzymes activities related to nitrogen metabolism were measured. NR activity was determined by measuring the nitrite produced from nitrate reduction using the diazotization reaction of nitrite with sulfanilamide and N-(1-naphthyl) ethylenediamine dihydrochloride, which forms a pink azo dye measurable at 540 nm and expressed by μmol·g^-1^·h^-1^. GS activity was assayed by the biosynthetic reaction, where the enzyme catalyzes the formation of γ-glutamylhydroxamate from glutamate and hydroxylamine; the hydroxamate forms a brown complex with ferric chloride under acidic conditions, measured at 540 nm, and expressed by A·g^-1^·h^-1^. GOGAT activity was measured by monitoring the oxidation of NADH at 340 nm, which is coupled to the reductive amination of α-ketoglutarate using glutamine as the amino donor, and the results was expressed by nmol·g^-1^·min^-1^.

### 2.8 Statistical analysis

In this study, Excel 2010 (Microsoft Corporation, Washington, USA) was used for the data processing and graph drawing. The SPSS 19.0 (IBM Corporation, New York, USA) was employed for data analysis with the least significant difference (LSD) test at the 5% probability level (*P < 0.05*). Principal component analysis (PCA) was supported by using MetaboAnalyst 6.0 (https://www.metaboanalyst.ca). The structural equation model (SEM) was constructed and validated by R studio (Posit Software, Massachusetts, USA) with matrix, nlme, lme4, piecewise SEM v2.1.2, and readx1 packages.

## 3 Results

### 3.1 Plant height, TFM, and TDM

**Table 1** showed that different concentrations of exogenous GABA applications exerted varying effects on plant height, TFM, and TDM of *I. indigotica* seedlings from both provenance areas at each sampling stage. For the Gansu provenance, the plant height reached its peaked value at 5d AF under T2 treatment, which was 4.21% higher than that of the CK. The maximum increases in TFM and TDM were observed at 3d AF under T2 treatment and 5d AF under T1 treatment, with increments of 12.79% and 6.90%, respectively. For the Hebei provenance, the highest plant height was recorded at 5d AF under T1 treatment. TFM and TDM showed maximum increases at 5d AF under T2 treatment and at 1d AF under T2 treatment, with increments of 12.62% and 4.48%, respectively. However, the inhibitory effects of the T3 treatment on plant height, TFM, and TDM were also observed in *I indigotica* seedlings from both provenance areas at all sampling stages, exceptions of TFM (1d AF) and TDM (1d AF and 5d AF) in the Gansu provenance, as well as TDM (1d AF) in the Hebei provenance.

**Table 1:** Effect of exogenous GABA application on plant height, TFM, and TDM of *I. indigotica* seedlings.

*I. indigotica* provenance area	Treatment	Plant height (cm)			TFM (g)			TDM (g)		
		1d AF	3d AF	5d AF	1d AF	3d AF	5d AF	1d AF	3d AF	5d AF
Gansu	CK	8.10 ± 0.13a	8.28 ± 0.11a	8.78 ± 0.11ab	2.81 ± 0.03b	2.97 ± 0.11b	3.34 ± 0.10ab	0.77 ± 0.01b	0.84 ± 0.01a	0.87 ± 0.00c
	T1	8.18 ± 0.09a	8.65 ± 0.16a	9.05 ± 0.23a	2.94 ± 0.06ab	3.14 ± 0.11ab	3.51 ± 0.10a	0.80 ± 0.01a	0.86 ± 0.01a	0.93 ± 0.01a
	T2	8.23 ± 0.08a	8.43 ± 0.13a	9.15 ± 0.18a	2.97 ± 0.03a	3.35 ± 0.15a	3.44 ± 0.08ab	0.78 ± 0.01ab	0.86 ± 0.01a	0.91 ± 0.01b
	T3	8.08 ± 0.13a	8.23 ± 0.11a	8.45 ± 0.14b	2.90 ± 0.03ab	2.95 ± 0.07b	3.16 ± 0.08b	0.79 ± 0.00ab	0.81 ± 0.01b	0.88 ± 0.01c
Hebei	CK	7.60 ± 0.18ab	7.63 ± 0.20b	7.83 ± 0.06b	2.61 ± 0.03b	2.84 ± 0.04bc	3.25 ± 0.08b	0.67 ± 0.01b	0.71 ± 0.00b	0.79 ± 0.01a
	T1	7.90 ± 0.04a	8.05 ± 0.10ab	8.33 ± 0.09a	2.75 ± 0.04a	2.94 ± 0.05ab	3.38 ± 0.04b	0.68 ± 0.01ab	0.74 ± 0.01a	0.80 ± 0.01a
	T2	7.98 ± 0.11a	8.23 ± 0.18a	8.30 ± 0.20a	2.74 ± 0.04a	3.00 ± 0.05a	3.66 ± 0.08a	0.70 ± 0.01a	0.72 ± 0.00b	0.81 ± 0.01a
	T3	7.38 ± 0.19b	7.55 ± 0.17b	7.60 ± 0.13b	2.58 ± 0.04b	2.75 ± 0.04c	2.99 ± 0.04c	0.68 ± 0.00ab	0.69 ± 0.00c	0.75 ± 0.01b

The different lowercase letters above the bars indicate significant differences between treatments applied to *I. indigotica* plants of the same provenance by LSD tests at *P*<0.05. CK, T1, T2, and T3 represent spraying 0, 2.5, 5, and 7.5 mM·L-1 of GABA at the six-leaf stage. AF, after spraying; TFM, Total Fresh Mass; TDM, Total Dry Mass.

### 3.2 Activities of SOD, POD, and CAT

**Figure 1:**
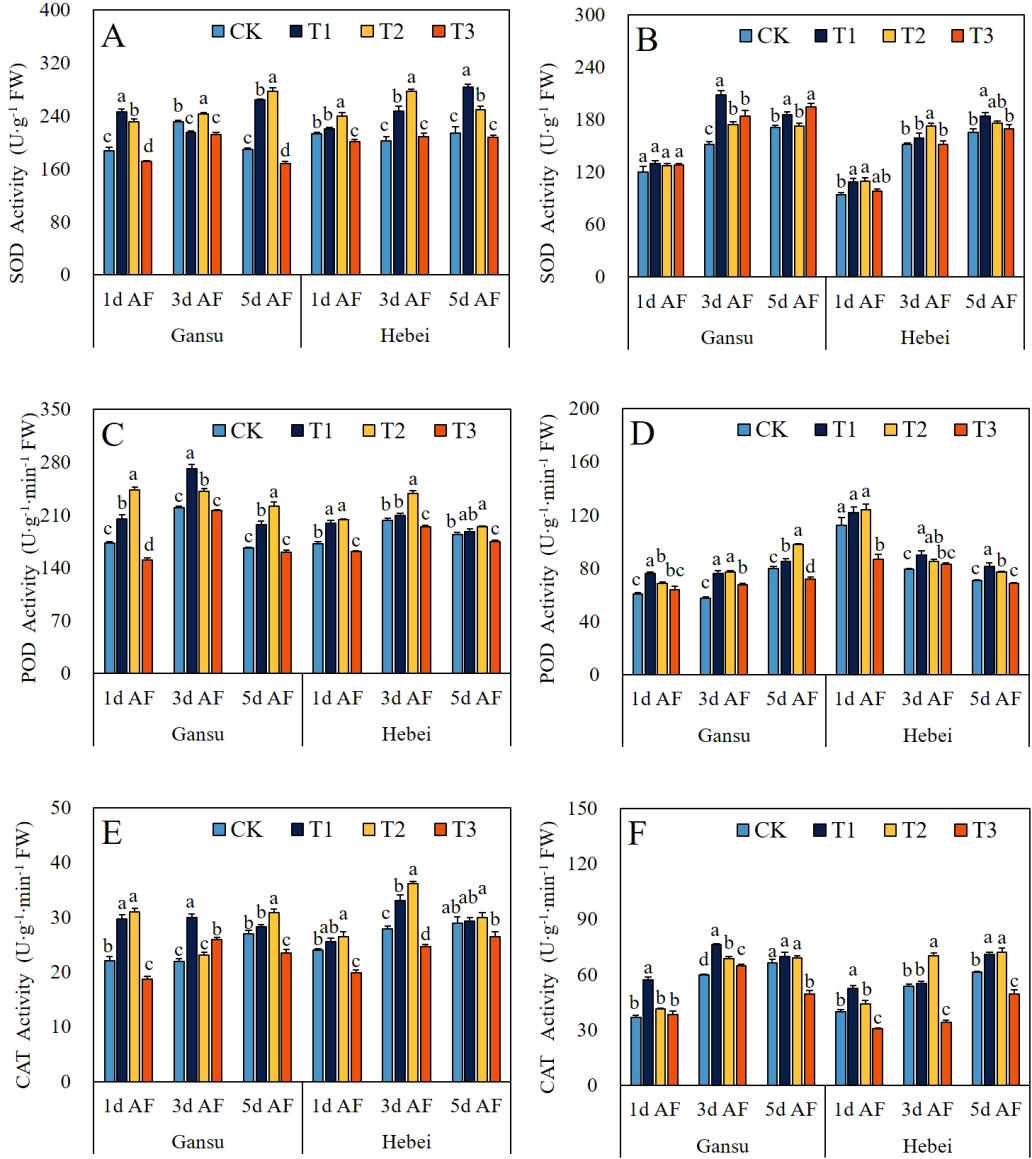
Effect of exogenous GABA application on the activities of SOD **(A, B)**, POD **(C, D)**, and CAT **(E, F)** in leaves and roots of *I. indigotica* seedlings at different sampling stages. The different lowercase letters above the bars indicate significant differences between treatments applied to *I. indigotica* plants of the same provenance by LSD tests at *P*<0.05. CK, T1, T2, and T3 represent spraying 0, 2.5, 5, and 7.5 mM·L^–1^ of GABA at the six-leaf stage. SOD, Superoxide dismutase; POD, Peroxidase; CAT, Catalase.

Compared with CK, the activities of SOD, POD and CAT in leaves and roots of *I. indigotica* seedlings from both provenance areas at different sampling stages were regulated by exogenous GABA application at different concentrations (**Figure 1**). For the Gansu provenance, T1 and T2 treatments improved the activities of SOD, POD and CAT increased in both leaves and roots, except the activity of SOD at 3d AF under T1 treatment. The highest SOD activities in leaves and roots were observed under T2 treatment at 5d AF and T1 treatment at 3d AF, with increments of 45.65% and 37.34%, respectively. The POD activity in leaves and roots was significantly increased by T1 and T2 treatments at all sampling stages. Additionally, the CAT activity in leaves and roots was maximally increased by 40.46% under T2 treatment at 1d AF and by 28.02% under T1 treatment at 3d AF, respectively. For the Hebei provenance, the maximum values of SOD, POD, and CAT activities in leaves and roots were recorded at 5d AF under T1 treatment, 3d AF under T2 treatment, and 5d AF under T2 treatment, which were increased by 32.66%, 17.24%, and 41.02%, respectively.

### 3.3 Contents of soluble protein contents, H_2_O_2_, and MDA

**Figure 2:**
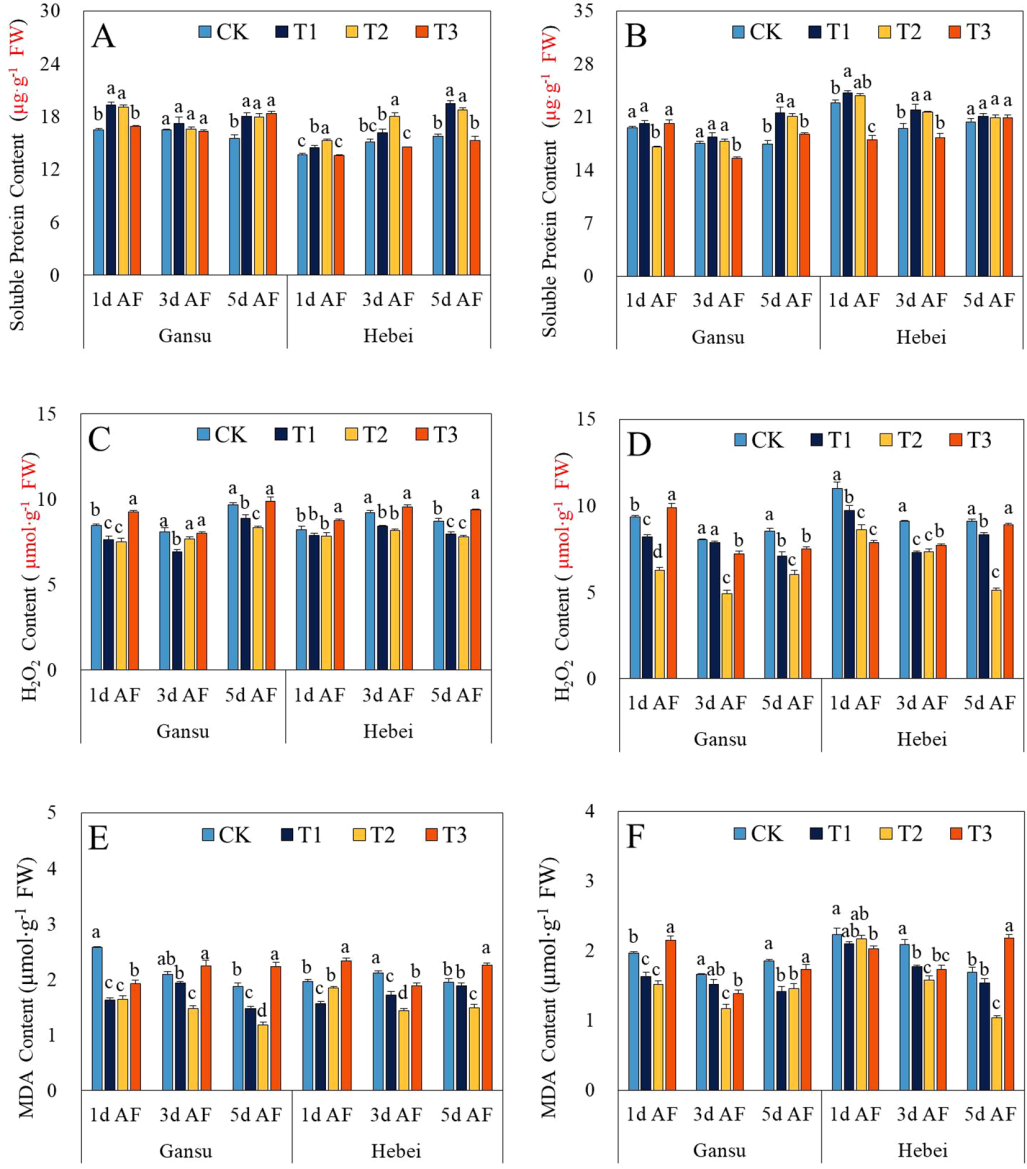
Effect of exogenous GABA application on the contents of soluble protein **(A, B)**, H_2_O_2_**(C, D)**, and MDA **(E, F)** in leaves and roots of *I. indigotica* seedlings at different sampling stages. The different lowercase letters above the bars indicate significant differences between treatments applied to *I. indigotica* plants of the same provenance by LSD tests at *P*<0.05. CK, T1, T2, and T3 represent spraying 0, 2.5, 5, and 7.5 mM·L^−1^ of GABA at the six-leaf stage. H_2_O_2_, Hydrogen peroxide; MDA, Malondialdehyde.

As shown in **Figures 2A, B**, the promotion of soluble protein content in leaves and roots of *I. indigotica* seedlings from two provenance areas was discovered at all sampling stages induced by T1 and T2 treatments, except for the roots of the Gansu provenance at 1d AF under T2 treatment. Conversely, T1 and T2 treatments reduced the contents of MDA and H_2_O_2_ at each sampling stage. The minimum values of these two indicators were recorded respectively in the roots of Hebei provenance under T2 treatment at 5d AF and in the roots of Gansu provenance under T2 treatment at 3d AF (**Figures 2C–F**).

### 3.4 Activities of NR, GOGAT, and GS

**Figure 3:**
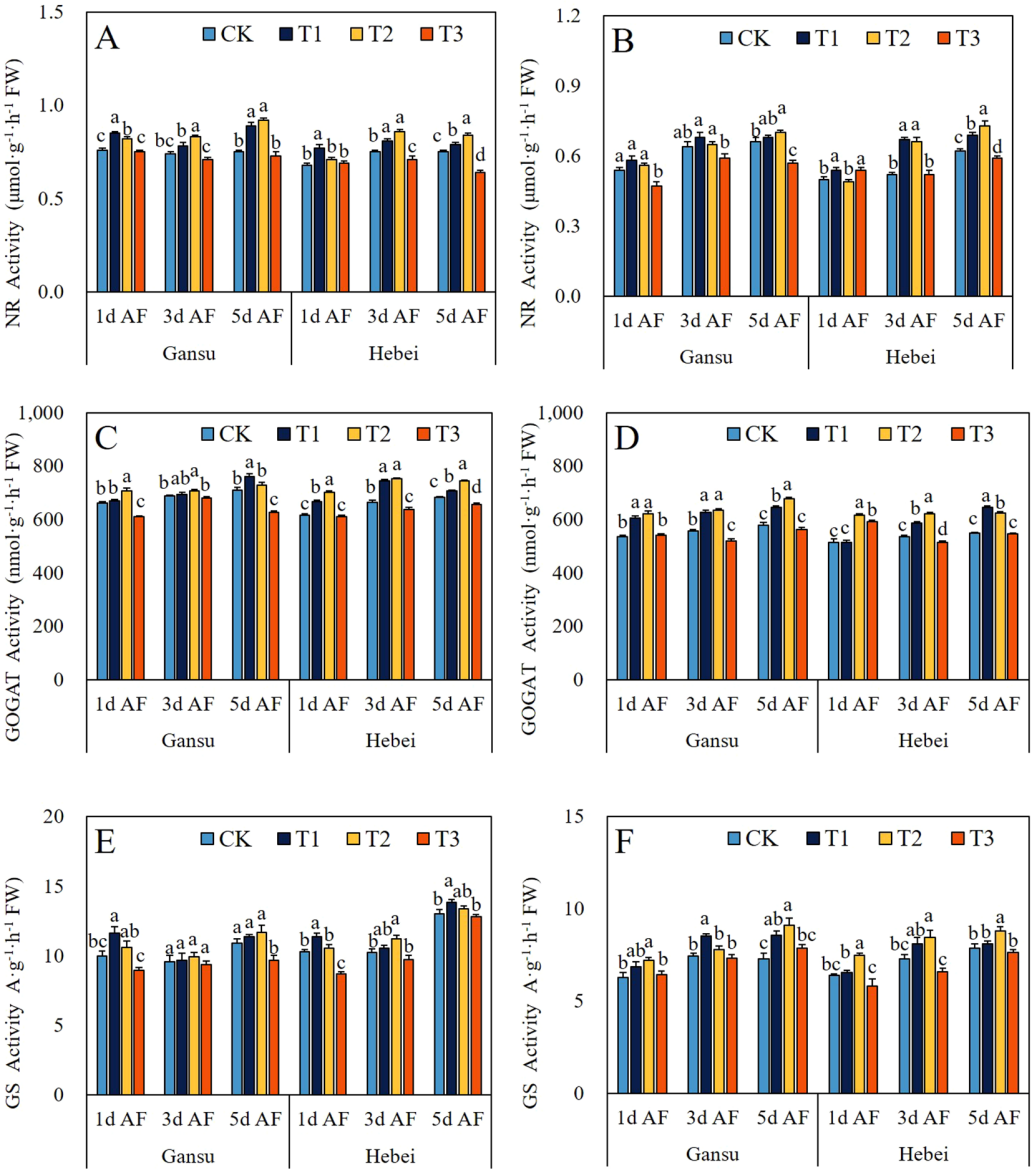
Effect of exogenous GABA application on the nitrogen metabolism enzymes activities of NR **(A, B)**, GOGAT **(C, D)**, and GS **(E, F)** in leaves and roots of *I. indigotica* seedlings at different sampling stages. The different lowercase letters above the bars indicate significant differences between treatments applied to *I. indigotica* plants of the same provenance by LSD tests at *P*<0.05. CK, T1, T2, and T3 represent spraying 0, 2.5, 5, and 7.5 mM·L^−1^ of GABA at the six-leaf stage. NR; Nitrate reductase; GOGAT, Glutamate synthase; GS, Glutamine synthetase.

As shown in **Figure 3**, T1 and T2 treatments increased the activities of NR, GOGAT, and GS in leaves and roots of *I. indigotica* seedlings from both provenance areas at all sampling stages, with the only exception being root NR activity in the Hebei provenance under T2 treatment at 1d AF. For the Gansu provenance, the NR and GS activities in leaves and roots reached the highest values at 3d AF under T2 treatment, and the highest GOGAT activities in leaves and roots were recorded at 3d AF with T1 and T2 treatments, respectively. For the Hebei provenance, the maximum activities of NR, GS, and GOGAT in leaves were recorded at 3d AF under T2 treatment, 5d AF under T1 treatment, and 3d AF under T2 treatment, respectively. In contrast, the activities of these three enzymes in roots all peaked at 3d AF, with maximum values observed under T2 treatment for NR and GS, and under T1 treatment for GOGAT.

### 3.5 Photosynthetic capacity

**Figure 4:**
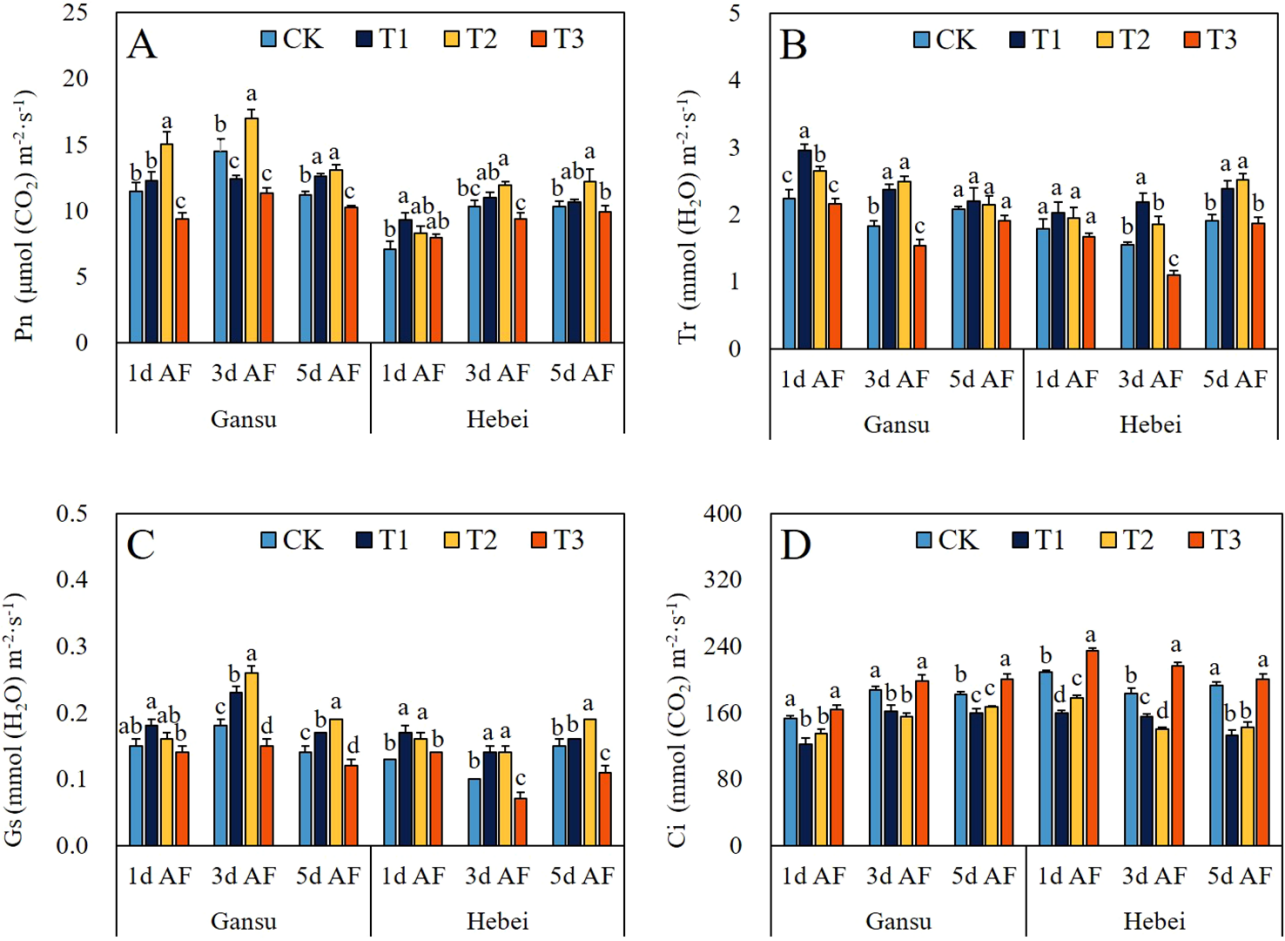
Effect of exogenous GABA application on the Pn **(A)**, Tr **(B)**, Gs **(C)**, and Ci **(D)** in *I. indigotica* seedlings at different sampling stages. The different lowercase letters above the bars indicate significant differences between treatments applied to *I. indigotica* plants of the same provenance by LSD tests at *P*<0.05. CK, T1, T2, and T3 represent spraying 0, 2.5, 5, and 7.5 mM·L^−^¹ of GABA at the six-leaf stage. Pn, Net photosynthetic rate; Tr, Transpiration rate; Gs, Stomatal conductance; Ci, Intercellular CO_2_ concentration.

Compared to CK, the exogenous GABA application could regulate the parameters related to the photosynthetic capacity (**Figure 4**). For *I. indigotica* seedlings from both provenance areas, the highest values of Pn and Gs were observed at 3d AF under T2 treatment, with respective increases of 17.55% and 44.44%, respectively (**Figures 4A, C**). The maximum Tr value was recorded at 1d AS under T1 treatment (**Figure 4B**). However, T1 and T2 treatments dramatically reduced the Ci, whereas the T3 treatment increased the Ci at each sampling stage (**Figure 4D**).

### 3.6 Root morphology

**Figure 5:**
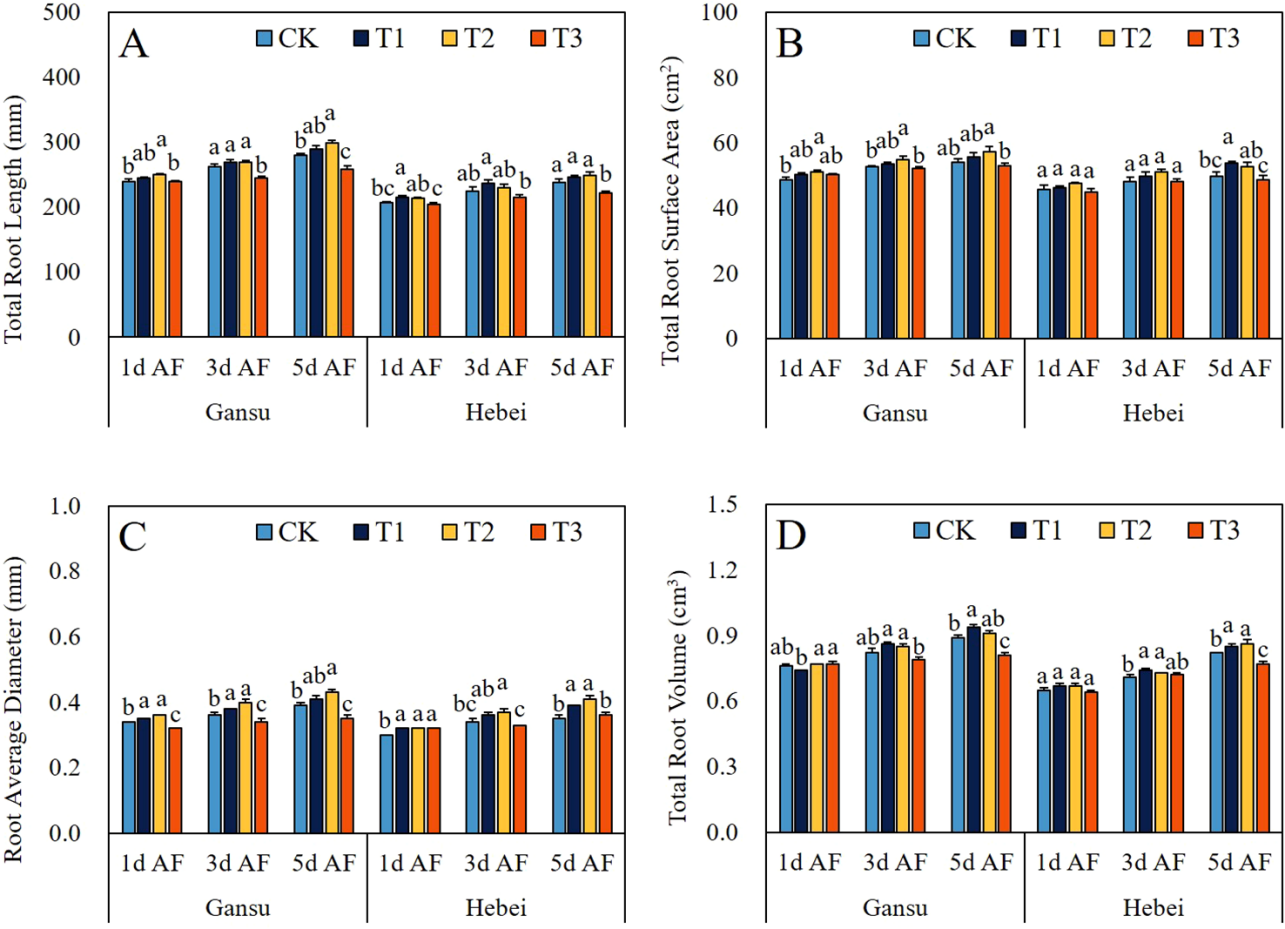
Effect of exogenous GABA application on the root total length **(A)**, root total surface area **(B)**, root average diameter **(C)**, and root total volume **(D)** in *I. indigotica* seedlings at different sampling stages. The different lowercase letters above the bars indicate significant differences between treatments applied to *I. indigotica* plants of the same provenance by LSD tests at *P* < 0.05. CK, T1, T2, and T3 represent spraying 0, 2.5, 5, and 7.5 mM·L^−1^ of GABA at the six-leaf stage.

The modulation of root morphology induced by exogenous GABA application was supported by **Figure 5**. The total root length, root average diameter, and total root volume all peaked in the T2 treatment at 5d AF for both provenance areas of *I. indigotica* seedlings (**Figures 5A, C, D**). At the same sampling stage, the highest total root surface area was observed in the Hebei provenance under the T1 treatment and the Gansu provenance under the T2 treatment, with respective increases of 8.00% and 6.03% compared to the control (CK) (**Figure 5B**).

### 3.7 Contents of pigment

**Figure 6:**
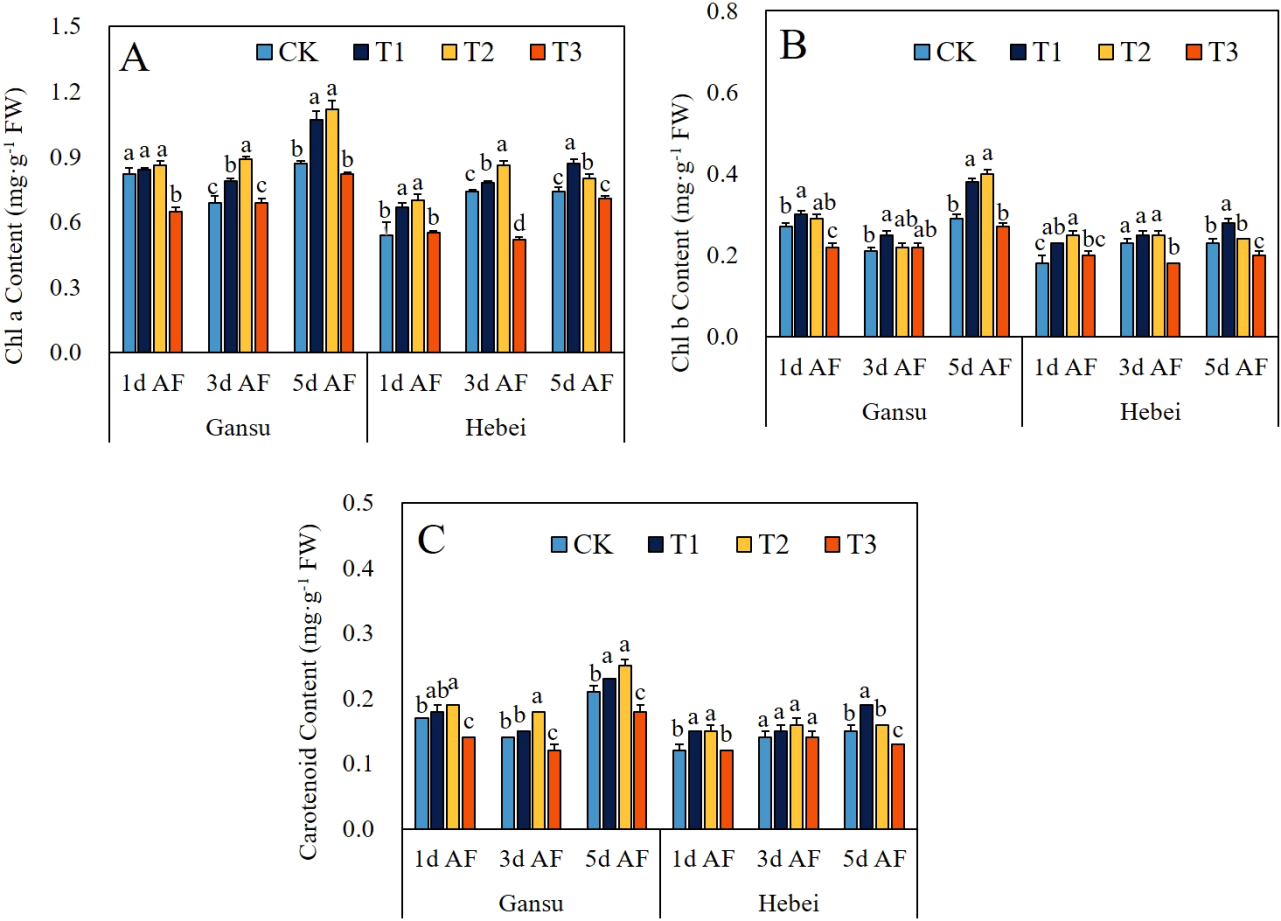
Effect of exogenous GABA application on the contents of Chl a **(A)**, Chl b **(B)**, and carotenoid **(C)** in leaves of *I. indigotica* seedlings at different sampling stages. The different lowercase letters above the bars indicate significant differences between treatments applied to *I indigotica* plants of the same provenance by LSD tests at *P* < 0.05. CK, T1, T2, and T3 represent spraying 0, 2.5, 5, and 7.5 mM·L^−1^ of GABA at the six-leaf stage. Chl a, Chlorophyll a; Chl b, Chlorophyll b.

The effects of exogenous GABA application on pigment contents were illustrated in **Figure 6**. The Chl a content exhibited the greatest increase in the Gansu provenance under the T2 treatment at 5d AF and in the Hebei provenance under the T2 treatment at 1d AF, with increments of 28.42% and 28.18%, respectively (**Figure 6A**). For Chl b and carotenoid contents, peak values were both detected at 5d AF, specifically, in the Gansu provenance under the T2 treatment and in the Hebei provenance under the T1 treatment (**Figures 6B, C**).

### 3.8 Direct and indirect regulation of exogenous GABA application on total biomass accumulation, plant height, and root morphology in *I. indigotica* seedlings

**Figure 7:**
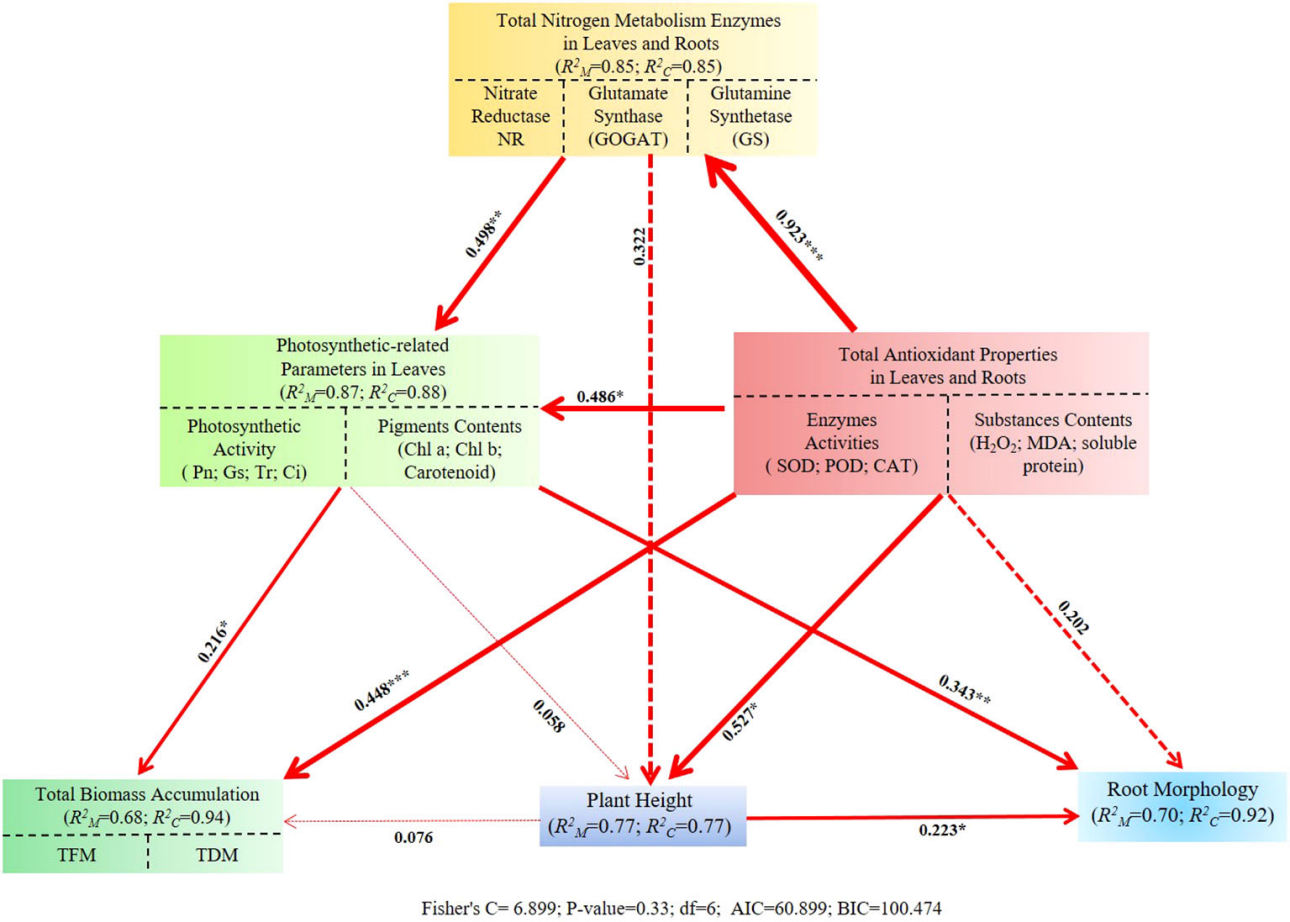
The Structural equation modeling (SEM) revealed the direct and indirect effects of the relevant parameters on the growth characteristics of *I. indigotica* seedlings. NR, nitrate reductase; GOGAT, glutamate synthase; GS, glutamine synthetase; Pn, net photosynthetic rate; Gs, stomatal conductance; Tr, transpiration rate; Ci, intercellular CO_2_ concentration; Chl a, chlorophyll a; Chl b, chlorophyll b; SOD, superoxide dismutase; POD, peroxidase; CAT, catalase; H_2_O_2_, hydrogen peroxide; MDA, malondialdehyde; TFM, total fresh mass; TDM, total dry mass. The value marked near each arrow and the thickness of the arrow indicate the direct effect coefficient in the path and the correlation degree between the two modules, respectively. *P < 0.05 **P < 0.01 ***P < 0.001.

To clarify the direct and indirect effects of total antioxidant properties and total nitrogen metabolism enzyme activities in leaves and roots, photosynthetic-related parameters in leaves, and plant height on total biomass accumulation and root morphology under different concentration exogenous GABA application, a SEM was constructed and validated. According to the results analyzed by SEM, the photosynthetic-related parameters in leaves and total antioxidant properties in leaves and roots played a dominant role in regulating total biomass accumulation (*R^2M^* = 0.68), and the photosynthetic-related parameters in leaves and plant height accounted for the major direct effects on the root morphology (*R^2M^* = 0.70). The total antioxidant properties in leaves and roots exerted a significant positive influence on the total nitrogen metabolism enzyme activities in leaves and roots, photosynthetic-related parameters in leaves and plant height with path coefficients of 0.923, 0.486, and 0.527, respectively. Notably, the total nitrogen metabolism enzyme activities in leaves and roots could indirectly regulate the total biomass accumulation and root morphology by exerting a significant direct effect on the photosynthetic-related parameters in leaves with a corresponding path coefficient of 0.498 (**Figure 7**).

### 3.9 PCA analysis

**Figure 8:**
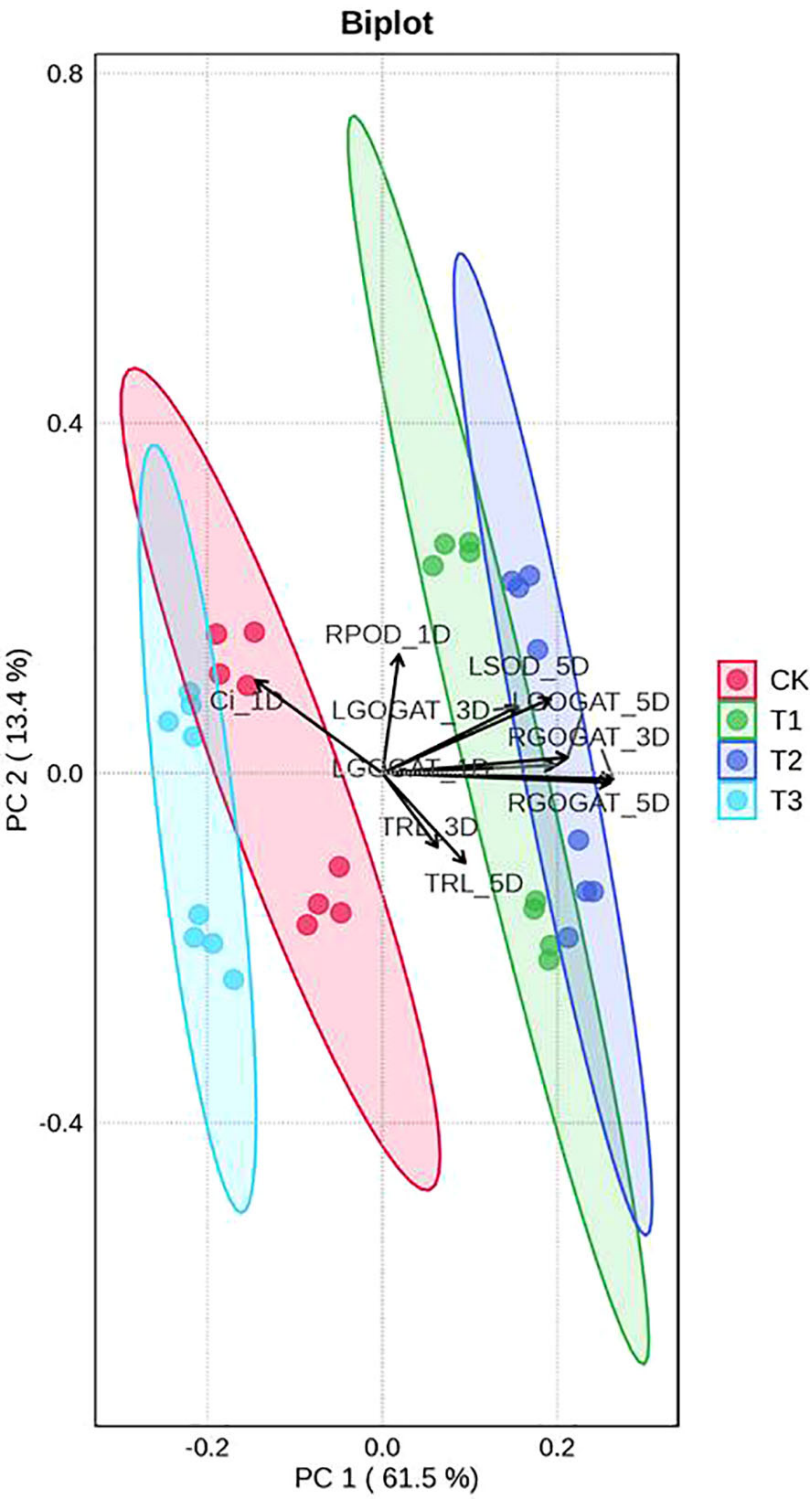
Principal component analysis (PCA) biplot between the samples of two *I. indigotica* seedlings under different treatments. CK, T1, T2, and T3 represent spraying 0, 2.5, 5, and 7.5 mM·L^−1^ of GABA at the six-leaf stage. GOGAT, glutamate synthase; SOD, superoxide dismutase; POD, peroxidase; TRL, total root length; Ci, intercellular CO_2_ concentration; R, root; L, leaf; D, days after spraying.

The PCA biplot captured 74.9% of total variance across treatments, with PC1 and PC2 accounting for 61.5% and 13.4% of variability, respectively (**Figure 8**). This analysis revealed distinct treatment-driven patterns: CK and T3 clustered in the second, third and fourth quadrants, while T1 and T2 occupied the first, second and fourth quadrants. Critically, T2 treatment showed a marked spatial separation from CK, with its data points localized in the first and fourth quadrants-suggesting substantial divergence in seedling traits regulated by exogenous appropriate concentrations GABA versus the control. Along PC1, the strongest correlations aligned with GOGAT activities in both leaves and roots, highlighting central roles of this enzyme in driving primary variance. For PC2, the dominant contributor was root POD activity at 1d post-spraying, underscoring a time-specific, root-centered physiological response modulated by exogenous GABA. Collectively, these PCA patterns elucidate how exogenous GABA treatment reshapes key biochemical signatures (GOGAT, POD) and spatial clustering of seedling traits, which can provide mechanistic insights into GABA-mediated regulation of *I. indigotica* seedling quality.

## 4 Discussion

Since the O_2_ involved in aerobic metabolism in the form of reduction or activation, reactive oxygen species (ROS) have been an inevitable by-product of metabolism throughout the entire lives of plants and animals (Choudhury et al., 2017). During the continuous evolutionary process of plants, higher plants have acquired a series of complex and specific antioxidant enzymes and substances to eliminate the damage caused by ROS (Das and Roychoudhury, 2014). Hence, the enzymes activities (containing CAT, POD, and SOD) and the contents of soluble protein, MDA, and H_2_O_2_ were detected in this study, which evaluated the modulation of antioxidant properties in *I. indigotica* seedlings treated by exogenous GABA application. Compared with CK, the SOD, POD, and CAT activities and soluble protein content were improved, while the MDA and H_2_O_2_ contents in leaves and roots of *I. indigotica* seedlings from both provenance areas were decreased by T1 and T2 treatments at each sampling stage (**Figures 1**, **2**) The only exceptions were the soluble content in roots from Gansu provenance treated with T2 at 1d AS and the SOD activity in leaves from Gansu provenance treated with T1 at 3d AS (**Figures 1**, **2**). Those results indicated the exogenous GABA application could strengthen the antioxidant capacity of *I. indigotica* seedlings via promoting cell membrane stability, enhancing the levels of non-enzymatic and enzymatic antioxidants, and reducing the generation of ROS. Meanwhile, these findings are consistent with those of several previous studies (Li et al., 2016; Shelp et al., 2021; Aljuaid and Ashour, 2022; Ahmad and Fariduddin, 2025; Geng et al., 2025).

The process of photosynthesis is when green plants capture and absorb light energy, then use it to biosynthesize organic carbon compounds by assimilating CO_2_ and H_2_O, while releasing O_2_ into the surrounding environment (Stirbet et al., 2020). Meanwhile, the regulation of plant photosynthetic capacity induced by exogenous GABA application has been reported by many previous studies. For example, Li et al (Das and Roychoudhury, 2014) discovered that exogenous application of 50 mg·L^-1^ GABA could result in a consistent elevation of Pn, Gs, and Tr in seedling populations of two distinct maize varieties at both experimental stages. Meanwhile, an enhancement of Pn, Gs, Ci, and Tr at the first sampling stage was documented by Yu et al. (2024) when exogenous treatment of sugar beet seedlings with 1.5 mM·L^-1^ GABA. Moreover, the promotional effect on photosynthetic capacity of other kinds of plants treated by exogenous GABA application has been reported by numerous studies (Sun et al., 2023; Zarbakhsh and Shahsavar, 2023; Ahmad and Fariduddin, 2025). In this study, the values of Pn, Gs, and Tr of *I. indigotica* seedlings were all increased under T1 and T2 treatments at each experimental stage, except for the Pn in Gansu provenance seedlings under T1 treatment at 3d AS (**Figure 4**). Those results could indicate that the foliar application of appropriate GABA concentration could promote the photosynthetic capacity of *I. indigotic.* Interestingly, Ci values decreased at each sampling stage under T1 and T2 treatments (**Figure 4**). This finding was similar to the paper published by Cheng et al. (2022) who demonstrated that the α-ketoglutaric acid foliar application could enhance the photosynthetic capacity due to the elevation of Pn, Gs, and Tr, coupled with a consistent consumption in intercellular CO_2_. Moreover, excluding the treatment with the highest concentration of GABA, all other GABA-treated groups exhibited increased pigment contents at each sampling stage (**Figure 6**). Our results agreed with the study of Aljuaid and Ashour (2022) who demonstrated that exogenous spraying of 0.5 mM·L^-1^ and 1 mM·L^-1^ GABA led to an elevation in chlorophyll a, chlorophyll b, and carotenoid contents in maize seedlings. Importantly, SEM analysis revealed not only a direct effect of total antioxidant properties in leaves and roots on photosynthetic-related parameters in leaves but also the presence of an indirect effect from total antioxidant properties in leaves and roots to photosynthetic-related parameters in leaves (**Figure 7**). These results provided additional reason to illustrate the enhanced photosynthetic capacity observed in *I. indigotica* seedlings under GABA treatment.

In addition, the total biomass accumulation capacities and plant height variations of *I. indigotica* seedlings treated by exogenous GABA application were also assessed in this presented study. Interestingly, at each sampling stage, T1 and T2 treatments increased the plant height, TFM, and TDM of *I. indigotica* seedlings from both provenance areas (**Table 1**). Those results might be caused by the following reasons: (1) with the facilitated effect of exogenous GABA application on plant photosynthetic capacity, the accumulation of photosynthetic products (including soluble sugar and sucrose) might be promoted. In turn, the elevated carbohydrate content further enhances plant height and biomass accumulation. In addition, an indirect effect from total antioxidant properties through photosynthetic-related parameters in leaves to TBM was supplied by SEM results (**Figure 7**). This hypothesis was supported by the study of Zarbakhsh and Shahsavar (2023) who demonstrated that exogenous application of 40 mM·L^-1^ GABA could improve photosynthetic capacity, sugar contents, and both fresh and dry weight of the root and aerial parts in pomegranate plants. (2) Besides its role in enhancing plant resistance to various stresses, GABA also functions as both a supplementary nitrogen source and a signaling molecule. Specifically, it contributes to the stabilization of carbon-nitrogen (C:N) balance and carbon/nitrogen (C/N) fluxes, while regulating nitrogen and carbon metabolism through multiple complex enzymatic pathways (Li et al., 2016; Shelp et al., 2021; Zarbakhsh and Shahsavar, 2023). In this study, across all experimental stages, T1 and T2 treatments enhanced the activities of NR, GS, and GOGAT in both roots and leaves of *I. indigotica* seedlings from both provenance areas. The sole exception was the NR activity in Hebei provenance seedlings subjected to T2 treatment at 1d AS (**Figure 3**). Meanwhile, the SEM results indicated that exogenous GABA application promoted the levels of total antioxidant properties in leaves and roots, regulated the total nitrogen metabolism enzyme activities in leaves and roots, and enhance photosynthetic activity and pigment contents, which ultimately resulted in total biomass accumulation increasement (**Figure 7**). In addition, PCA resulted the strongest correlation indicators with the PC1 were GOGAT activity in roots at 5d post-spraying (**Figure 8**). These results aligned with the finding of Li et al. (2016) who reported that the shoot dry weight under foliar application of 50 mg·L^-1^ GABA treatment was positively correlated with NR activity, Pn and Tr. (3) In SEM, two direct links were identified from total antioxidant properties in leaves and roots to both total biomass accumulation and plant height (**Figure 7**). Based on these findings, it was speculated that the enhancement of antioxidant properties induced by foliar application of GABA might increase total biomass accumulation and plant height by ensuring the integrity of cell membranes, continuity of metabolic pathways, and stability of physiological processes, all of which are essential for the growth and development of *I indigotica* seedlings. Furthermore, the plant height, TDM, and TFM of all provenance areas showed a decreasing trend under the T3 treatment at each experimental stage, except for the TFM of the Gansu provenance at 1d AF, the TDM of the Gansu provenance at 1d AF and 5d AF, and the TDM of the Hebei provenance at 1d AF (**Table 1**). These results agreed with the previous study by Yu et al. (2024) who demonstrated that the application of a higher concentration of GABA (specifically 5.0 mM·L^-1^) could inhibit the growth and development of sugar beet seedlings. The shift from promotion to inhibition with a relatively small increase in GABA concentration (from 5 mM to 7.5 mM) is consistent with a typical dose-response pattern observed for many plant growth regulators. This phenomenon likely indicates that the optimal concentration for enhancing physiological processes was exceeded, potentially leading to metabolic imbalance or feedback inhibition in key pathways such as nitrogen metabolism and antioxidant defense. Similar narrow windows between beneficial and inhibitory effects of exogenous GABA have been documented in other species, underscoring the importance of determining precise application concentrations for specific crops and growth conditions (Li et al., 2021; Song et al., 2025).

As an essential underground organ of plants, the root plays a pivotal role in multiple aspects of plant growth and development, with its core functions mainly reflected in the following three dimensions: (1) substance metabolism: including the absorption, exchange, transportation, storage, and biosynthesis of substances. (2) Mechanical support: beyond anchoring the plant stably in the soil, it also contributes to water and soil conservation. (3) Ecological processes: involving the competition, promotion, and complementarity among all coexisting organisms and intricate associations with soil microbes (Erktan et al., 2018; Freschet et al., 2021). Hence, the values of root morphological parameters can not only directly reflect the development status of the root system but also demonstrate its adaptability to various changes in the growth environment (Koevoets et al., 2016; Freschet et al., 2021). Over the past decades, numerous studies have demonstrated that exogenous application of GABA can regulate the growth and development of roots in various plant species. For instance, Yu et al. (2024) found that when 1.5 mM·L^-1^ GABA was applied under salt stress (300 mM·L^-1^ NaCl), the root growth morphological parameters of sugar beet seedlings reached optimal levels. Meanwhile, Zhou et al. (2021) reported a significant increase in the root length of white clover seedlings following priming with 2 μM·L^-1^ GABA. Meanwhile, several studies have revealed the regulatory effects of GABA application on root development in woody plants (Xie T. et al., 2020; Wang et al., 2021; Pei et al., 2022; Zarbakhsh and Shahsavar, 2023). In our study, both T1 and T2 treatments promoted all root morphological parameters of *I. indigotica* seedlings from two provenance areas, with the exception of the total root volume in Gansu provenance seedlings under T1 treatment at 1d AS (**Figure 5**). Additionally, the SEM analysis revealed two indirect pathways originating from total antioxidant properties in leaves and roots that affect root morphology, with each mediated by photosynthetic-related parameters in leaves and plant height, respectively (**Figure 7**). These results provided further evidence that exogenous GABA application could modify root growth and development.

## 5 Conclusions

In summary, T1 and T2 treatments (2.5 and 5 mM·L^-1^ GABA) improved the plant height and total biomass accumulation via an increase in photosynthetic capacity (Pn, Tr, and Gs), pigment contents, and nitrogen metabolism enzymes activities (NR, GS, and GOGAT) in *I. indigotica* seedlings of both provenance areas. Meanwhile, the root morphology was also promoted under T1 and T2 treatments. Conversely, T3 treatment (7.5 mM·L^-1^ GABA) showed an inhibition of the plant height, total biomass accumulation, and root morphology of *I. indigotica* seedlings. In addition, T1 and T2 treatments enhanced the antioxidant properties by increasing the activities of SOD, POD, and CAT, as well as soluble protein content, while reducing the accumulation of MDA and H_2_O_2_. Further analysis indicated that the enhancement of total antioxidant properties could regulate the plant height, total biomass accumulation, and root morphology through both direct and indirect pathways based on the structural equation mode. And the most significant divergence in seedling traits regulated by T2 treatment by PCA result. Overall, given the beneficial effects of exogenous GABA at optimal concentrations (5 mM·L^-1^) on the growth and development of *I. indigotica* seedlings, GABA has potential as a plant growth regulator for application in the cultivation of medicinal plants. To further elucidate the mechanisms by which exogenous GABA modulates the physiological and biochemical parameters of *I. indigotica* seedlings, additional studies are required at the molecular level and through multimodal omics analyses.

## Data Availability

The original contributions presented in the study are included in the article/supplementary material. Further inquiries can be directed to the corresponding author.
